# The Efficiency of TB LAM Antigen Test to Xpert MTB/RIF Ultra Test for the Diagnosis of Tuberculous Pericarditis Using Pericardial Fluid Samples

**DOI:** 10.3390/pathogens12091175

**Published:** 2023-09-19

**Authors:** Samuel Alomatu, Sandeep Vasaikar, Kandathil Thomas, Thozama Dubula, Khulile Moeketsi

**Affiliations:** 1 Cardiology Unit, Department of Internal Medicine, Nelson Mandela Central Hospital, Walter Sisulu University, Mthatha 5117, South Africa; kthomas@wsu.ac.za (K.T.); kmoeketsi@wsu.ac.za (K.M.); 2 Department of Microbiology, Faculty of Health Sciences, Walter Sisulu University, Mthatha 5100, South Africa; svasaikar@wsu.ac.za; 3 Office of Dean, Faculty of Health Sciences, Walter Sisulu University, Mthatha 5117, South Africa; tdubula@wsu.ac.za

**Keywords:** tuberculous pericarditis, Xpert MTB/RIF Ultra, TB LAM Ag, extrapulmonary tuberculosis, pericardial fluid, diagnosis

## Abstract

Medical considerations for early diagnosis of tuberculous pericarditis (TBP) include Xpert MTB/RIF Ultra and TB lipoarabinomannan (LAM) antigen (Ag) tests, with immunological status influencing the performance of the latter. An evaluation of the efficiency of Xpert MTB/RIF Ultra and TB LAM Ag in detecting TBP was conducted using pericardial fluid samples from 46 patients with suspected TBP. Fifteen patients (34.1%) were diagnosed with TBP according to culture results. TB LAM Ag’s sensitivity, specificity, positive predictive value (PPV), negative predictive value (NPV), positive likelihood ratio (PLR), and negative likelihood ratio (NLR) were 33.3%, 100%, 100%, 74.4%, 0, and 0.67, respectively. The sensitivity, specificity, PLR, NLR, PPV, and NPV of Xpert MTB/RIF Ultra were 80%, 93.1%, 11.6, 0.21, 85.7%, and 90%, respectively. There was an association observed between a positive TB LAM Ag test and HIV status. When compared to the Xpert MTB/RIF Ultra test, TB LAM Ag has lower accuracy for the detection of microbiologically proven tuberculous pericarditis, yet its usage in HIV-positive populations may be worth exploring. The TB LAM Ag assay is not the best first-line test for the diagnosis of tuberculous pericarditis, and it should be used in conjunction with other diagnostic tests.

## 1. Introduction

*Mycobacterium tuberculosis* (Mtb) is a pathogenic bacterium that causes tuberculosis (TB) in humans. This bacterium is spread via aerosol droplets, and infected people have a 5–10 lifetime chance of acquiring active TB [1]. Although the transmission rate is stated to be slow due to the long incubation period, people with active tuberculosis (TB) can infect 5–15 additional people in a year [1] and up to 19% of their household members in HIV-endemic areas [2]. The presence of specific factors, most notably a weaker immune system, as with HIV infection, increases the likelihood of contracting tuberculosis after contact with the pathogen [1]. This subset is 16 times (uncertainty interval: 14–18 times) more likely to become infected with tuberculosis than HIV-uninfected people [3]. 

In 2021, 10.6 million individuals had active tuberculosis, 6.7% of whom were HIV-positive. This rate is a nearly 5% increase over the 5.8 million cases recorded in 2020. In the same year, 1.6 million people died from tuberculosis [4]. Without anti-TB medication administration to interrupt the disease process, 45% of HIV-uninfected people and nearly all HIV-infected people will die from the disease [1]. The high burden of this infection, along with the recent increase in incidence, necessitates a drastic eradication approach to meet within the next seven years as sustainable development goal (SDG) target 3.3 [5]. 

Given that inhalation is the only route of transmission, the alveoli of the lungs and adjacent structures are the initial sites of infection, leading to pulmonary tuberculosis [6]. Infection, however, may occur in distant tissues and organs. Tuberculosis infection outside of the pulmonary parenchyma is collectively known as extrapulmonary TB [6] and accounts for 16% of all TB cases [7]. 

Tuberculous pericarditis, which is the infection under investigation in this study, is a type of extrapulmonary tuberculosis (EPTB). Tuberculous pericarditis (TBP) is relatively infrequent, accounting for only 1% of all TB and 1–2% of EPTB cases [8,9,10]. The occurrence of TBP is, however, associated with substantial morbidity, including cardiac tamponade and constrictive pericarditis and mortality [11]. These complications are exacerbated by delayed or missed diagnosis [11], thus prompting the need for rapid diagnostic approaches. Recent developments to aid in the early detection of TB are low-complexity nucleic acid amplification assays, such as the Xpert MTB/RIF Ultra test and point-of-care diagnostic approach, such as TB lipoarabinomannan (LAM) antigen (Ag) test [12]. These assays can detect Mtb in both pulmonary and extrapulmonary samples in HIV-infected and -uninfected persons [13,14]. 

The Xpert MTB/RIF Ultra was designed to improve Mtb detection sensitivity similar to standard culture using the GeneXpert platform [12,15] and concurrently, detects rifampicin resistance [12]. The assay targets the Mtb pathogen’s *rpoB* (RNA polymerase) gene as well as the insertion sequences IS1081 and IS6110 [12,16]. The turnaround time for findings is two hours, making it effective for early and quick TBP diagnosis [17]. Unlike its predecessor, the Xpert MTB/RIF, the Xpert MTB/RIF ultra has the distinct benefit of being able to diagnose TB and identify resistance in smear-negative and HIV-associated TB patients [14]. However, the diagnostic usefulness of the Xpert MTB/RIF Ultra for TBP has not been well investigated. Two studies on the subject have been published; the most recent was from a Belgian population, and the other was from a Chinese population. 

The findings from previous research carried out in the Belgian population showed that the Xpert MTB/RIF Ultra test was able to detect EPTB with a sensitivity of 90% and specificity of 91.9% in comparison to culture findings for smear-positive and negative samples [17]. These findings do not reflect its diagnostic performance in TBP because a large variety of extrapulmonary fluids were evaluated. In a second previous study conducted in a Chinese population, the Xpert MTB/RIF Ultra demonstrated a sensitivity of 52.5% compared to Xpert and Culture, which had sensitivities of 34% and 21.5%, respectively, in detecting TB on extrapulmonary specimens [18].

The TB LAM Ag test is a lateral flow assay that can inform of the presence of Mtb when it detects lipoarabinomannan, a polysaccharide found in the cell wall of the pathogen [19]. Metabolically active or degenerating bacterial cells release this lipopolysaccharide. Thus, it is often present in most people with active TB disease [19], and when the TB LAM Ag test TB infection state is used, infection is confirmed within 30 min [20]. Although the TB LAM Ag assay was designed for use with urine specimens [21,22], evaluation of its accuracy using pericardial fluid samples has previously been investigated in patients with suspected TBP [23]. In this South African population, the TB LAM Ag test showed a sensitivity of 11.6% (6.0–21.3%) and a specificity of 88% (70.0–95.8%) using pericardial fluid (PCF) samples [23]. The positive predictive value (PPV) and negative predictive value (NPV) were 72.7% and 26.6%, respectively, whereas the positive likelihood ratio (PLR) and negative likelihood ratio (NLR) were 0.9 and 1.0 in this South African population [23]. In HIV-infected people with CD4 < 100 cells/mm^3^, the sensitivity of the PCF LAM test increased from 5.1% to 27.3% [23]. This demonstrates that it has poor sensitivity but high specificity and a high likelihood of finding TBP in HIV-infected individuals. Indeed, sensitivity was greater in HIV-positive people than in HIV-negative people [23]. Despite its limited sensitivity, the fact that the TB LAM Ag test can identify Mtb in PCF samples from HIV patients is enticing. 

As a result, in the current environment with high HIV-co-infected TBP patients, the diagnostic performance of pericardial fluid TB LAM Ag may be superior to Xpert MTB/RIF Ultra. 

However, there have been no studies to date that investigated the diagnostic accuracy of TB LAM Ag to Xpert MTB/RIF Ultra for TBP using PCF in comparison to microbiological culture findings. 

The purpose of this study was to evaluate the efficiency of TB LAM Ag compared to that of Xpert MTB/RIF Ultra in the diagnosis of tuberculous pericarditis when testing PCF samples from patients with suspected TBP.

## 2. Materials and Methods

### 2.1. Study Design and Participants

The research comprised patients with large pericardial effusions, who were sent to Nelson Mandela Central Hospital for pericardiocentesis between July 2019 and July 2020. Males and females aged 12 and above are enrolled. The study excluded pregnant women, patients who started anti-TB therapy more than a week before pericardiocentesis, and children under the age of 12. The study was granted permission by Walter Sisulu University’s Health Ethics and Bio-Safety Committee (Protocol number: 034/2019). The study’s site also granted verbal approval. Before participation in the study, each patient provided written informed consent or assent. Blood samples were obtained from consenting participants for the HIV enzyme-linked immunosorbent assay (ELISA) test. 

### 2.2. Sample Collection, Handling, and Analysis

A baseline echocardiogram was performed to determine the quantity of PCF to determine if patients met the inclusion criteria. This was followed by pericardiocentesis to obtain 60 milliliters (mL) of PCF. The volume adheres to the minimum of 60 mls of PCF made use of in a previous study [24]. 

For the three investigations, different quantities of PCF were pipetted into three specimen jars: 10 mls for the Ultra Xpert MTB/RIF Ultra assay, 40 mL for culture, and 60 microliters (µL) for the TB LAM Ag test. The specimen jars of each participant were labelled with a research identification (ID) number.

#### 2.2.1. Culture

For *Mycobacterium tuberculosis* culture, 40 mL of PCF was delivered to the laboratory. Cultures were kept in an incubator for up to 42 days. The microbiological culture findings confirmed the diagnosis of TBP. The patients were then grouped as follows:

1. TBP confirmed patient: Mtb was detected in microbiological cultures of the PCF sample.

2. Non-TBP patient: Mtb was not detected in microbiological cultures of PCF. 

#### 2.2.2. Xpert MTB/RIF Ultra Test (Cepheid, Sunnyvale, CA, USA)

A laboratory staff member performed the test on each participant using the 10 mls PCF stored in the specimen jar for Xpert MTB/RIF Ultra testing. To identify AFB, Ziehl–Neelsen stain was used to make smears for direct microscopy. If the samples were clinically sterile, they were immediately processed. 

Nonsterile samples were decontaminated and digested with N-acetyl-L-cysteine and sodium hydroxide (NALC/NaOH) method. After decontamination, a buffer comprising sodium hydroxide and isopropanol was added, and the mixture was incubated for 15 min at room temperature. 

The digested samples were then placed into the GeneXpert^®^ MTB/RIF Ultra instrument using version G4.0 cartridges. Each GeneXpert MTB/RIF Ultra cartridge was labelled with the participant ID provided to it. In the GeneXpert MTB/RIF Ultra instrument, the cartridges were spun. Using gene sequencing, discrepant rifampicin (RMP) resistance results were validated. The results were available three hours after they were collected.

#### 2.2.3. TB LAM Ag Test (Alere Determined TB LAM Ag test, Abbott Laboratories, Chicago, IL, USA)

The 60 µL PCF from the specimen jar was placed on the individual Alere Determine™ TB LAM Ag test strip. The test was performed as a point-of-care service by skilled professionals immediately in the echocardiography room. After applying the PCF specimen to the strip for 25 min, they were visually evaluated. The intensity of any visible band on the test strip was graded by comparing it to the intensities of the bands on a reference card provided by the manufacturer. The reference bands are divided into four grades (Grades 1–4, 4 = maximum intensity positivity), with all Grades 1–4 reporting lipoarabinomannan positivity. 

##### Statistical Analysis

The efficiency of the Xpert MTB/RIF Ultra and TB LAM Ag assays were determined by calculating the test’s sensitivity, specificity, PPV, NPV, PLR, and NLR using the Statistical Package for Social Sciences, IBM SPSS version 24 (SPSS Inc., Chicago, IL, USA). The sensitivity and specificity of both tests were further stratified according to HIV status, CD4 cell count, and age group. Non-numerical data were represented using counts and proportions, whereas numerical data were expressed using means and standard deviations. 

The proportion of TBP found using the TB LAM Ag, Xpert MTB/RIF Ultra and culture tests were computed using the number of positive laboratory findings for each test.

The detection rates for each test were calculated by comparing the proportion of positive and negative findings between one of the investigative tests and the reference test. The proportion of false positive and false negative results was displayed and further related to participant characteristics. The detection rate between the two investigation tests was also computed to understand the behavior of one test relative to the other. To examine if HIV status had any influence on all three investigations, a chi-square test was performed. A statistically significant difference was set at a *p*-value less than 0.05 for all inferential statistics.

## 3. Results

### 3.1. Interpretation of Results

#### 3.1.1. Characteristics of the Study Population

The researchers collected 46 PCF samples from 24 males and 22 females (median age: 48 years). The youngest participant was 14 years old, while the oldest was 80 years old. The HIV status of 43.4% (20/46) of the individuals was positive (median CD4+ T cell count 253 cells/mm^3^; mean viral load 2960 copies/mL) and 39.1% (n = 18/46) negative. Eight participants (8; 17.4%) declined the ELISA test; thus their HIV status was unknown. Table 1 shows the demographic and clinical attributes of the participants.

#### 3.1.2. TBP Detection Rates

Two participants’ TB LAM Ag test results were missing. The diagnostic efficiency analysis was performed using data from 44 participants. Of the 44 patients, 15 (34.1%) were diagnosed with TBP by culture, whereas 29 (65.9%) had no TBP. The final diagnosis for participants with non-TBP was not examined. The rate of TBP was significantly (*p* = 0.008) higher in participants with a CD4+ T cell count of less than 200 cells/mm^3^. The rates were also higher in participants older than 45 years (46.2%), HIV-positive (35%) and those with undetectable viral load (75%). These rates are presented in Table 2.

##### The Detection Rate of PCF Xpert MTB/RIF Ultra in Comparison to PCF Culture

The Xpert MTB/RIF Ultra test was positive in 14 (31.8%) samples and negative in 30 (68.2%). In comparison to culture results, PCF Xpert MTB/RIF Ultra produced two false positive cases and three false negative cases (Table 3).

All three false negative Xpert MTB/RIF results came from females, two of whom were HIV-positive (median CD4 count 344 cells/m^3^) and one of unknown HIV status, with an average age of 55.6 years. The two false positive Xpert MTB/RIF results obtained were also from females; one was HIV-infected (CD4 count 218 cells/m^3^, viral load 61 copies/mL) and the other uninfected. The average age for the two was 56.5 years.

##### The Detection Rate of PCF TB LAM Ag in Comparison to PCF Culture

The PCF TB LAM Ag test for Mtb detection was positive in 5 participants (11.4%) and negative in 39 (88.6%). The PCF TB LAM Ag provided 10 false negatives but no false positive results (Table 3). There were five men, five females, six HIV-negatives, three HIV-positives (mean CD4 count 277 cells/m^3^), and one participant with unknown HIV status among the false negatives. The subpopulation’s average age was 53.8 years. 

**Figure 1:**
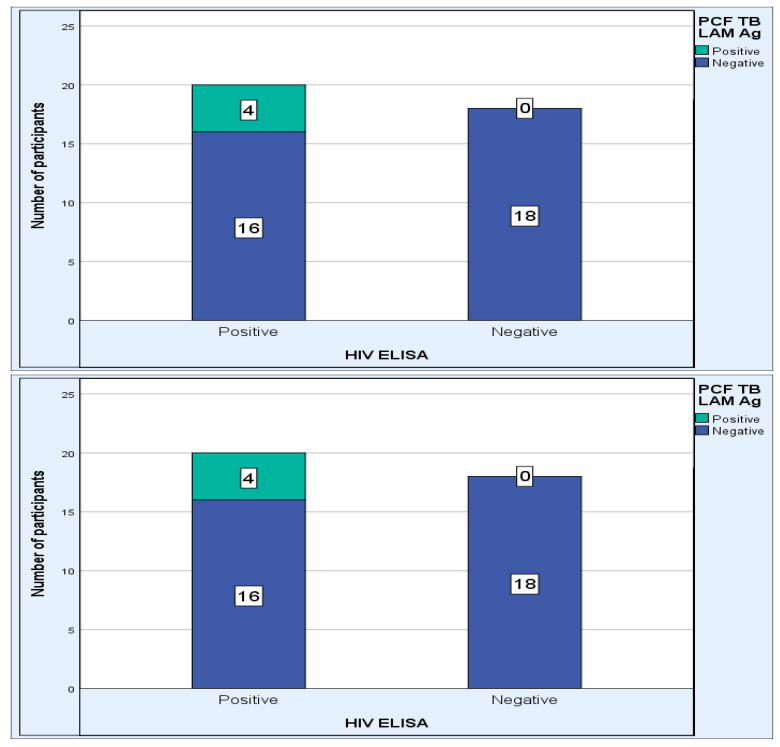
Relationship between HIV test result and PCF TB LAM Ag (n = 38).

Further analysis showed that the PCF TB LAM Ag results were not dependent (Fisher Exact *p* = 0.107) on the HIV Elisa results of the participant, although the PCF TB LAM Ag positive results occurred solely in HIV-positive participants (Figure 1).

##### The Detection Rate of PCF TB LAM Ag in Comparison to PCF Xpert MTB/RIF Ultra

**Figure 2:**
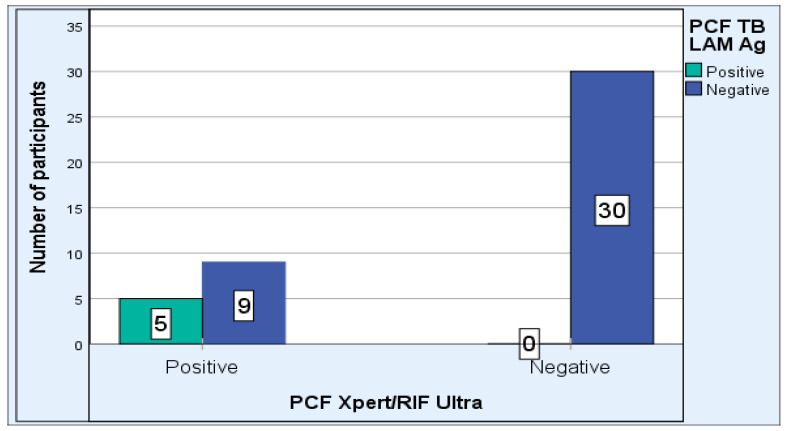
PCF Xpert MTB/RIF Ultra versus PCF TB LAM Ag results (n = 44).

All five PCF TB LAM Ag positive results were also positive for PCF Xpert MTB/RIF. Nine PCF TB LAM Ag negative results were positive with PCF Xpert MTB/RIF ultra. Xpert MTB/RIF Ultra detected more (n = 14) TBP cases, while the TB LAM Ag test produced more (n = 39) negative results (Figure 2).

#### 3.1.3. Diagnostic Efficiency

The tests’ diagnostic accuracy was measured and compared to that of microbiological culture. Table 4 summarizes the results obtained.

##### Xpert MTB/RIF Ultra Test

**Figure 3:**
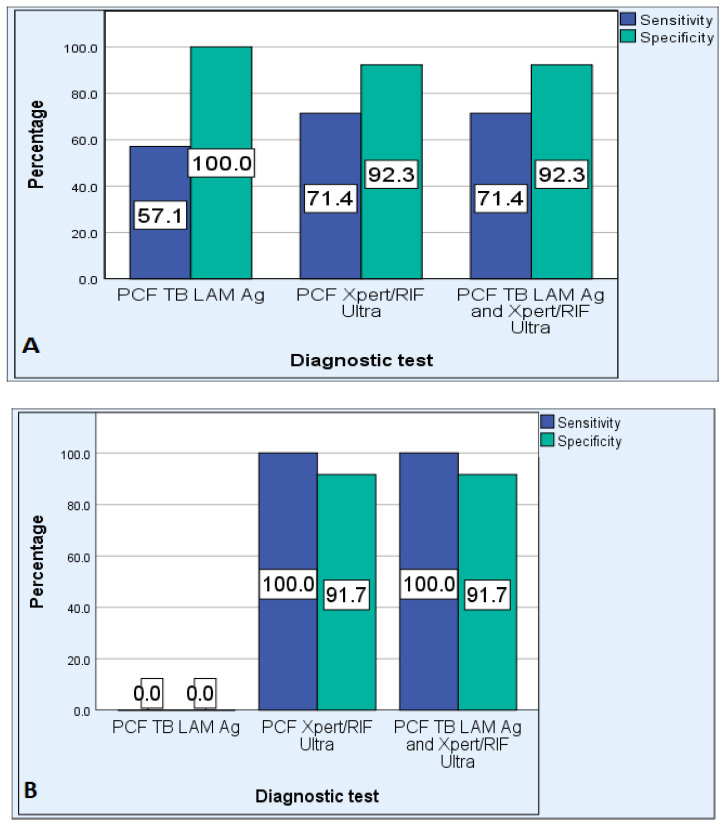
Performance of PCF TB LAM Ag, PCF Xpert MTB/RIF Ultra, and combination of PCF TB LAM Ag and PCF Xpert MTB/RIF Ultra assays in HIV-positive (**A**) and HIV-negative (**B**) cases.

**Figure 4:**
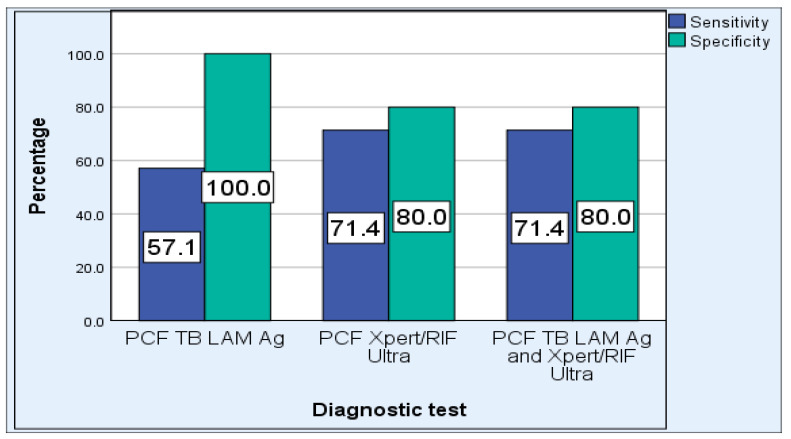
Performance of PCF TB LAM Ag, PCF Xpert MTB/RIF Ultra, and combination of PCF TB LAM Ag and PCF Xpert MTB/RIF Ultra assays in cases with CD4 count of less than 500 cells/mm^3^.

**Figure 5:**
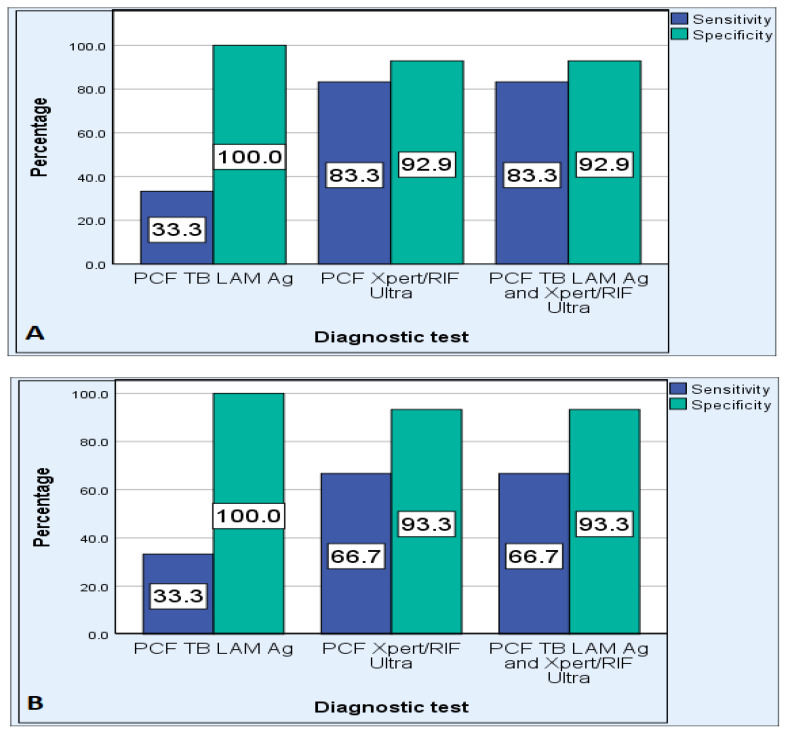
Performance of PCF TB LAM Ag, PCF Xpert MTB/RIF Ultra, and combination of PCF TB LAM Ag and PCF Xpert MTB/RIF Ultra assays in participants older than 45 years (**A**) and participants 45 years and younger (**B**).

Assessment of the overall efficiency of the Xpert MTB/RIF assay revealed that it has a sensitivity, specificity, PPV, NPV, PLR, and NLR of 80%, 93.1%, 85.7%, 90.0%, 11.6 and 0.21, respectively. The sensitivity of Xpert MTB/RIF ultra was higher in HIV-negative participants: 100% compared to HIV-positive (71.4%) (Figure 3). The specificity of Xpert MTB/RIF ultra was reduced to 80% in participants with a CD4 cell count of 500 cells/mm^3^ and less (Figure 4). The sensitivity of Xpert MTB/RIF ultra was higher in participants older than 45 years: 83.3% compared to younger participants (66.7%) (Figure 5). 

##### TB LAM Ag Test

The overall diagnostic sensitivity of the TB LAM Ag test using PCF was 33.3%, specificity was 100%, NPV was 74.4%, and PPV was 100%. The TB LAM Ag test had a PLR of 0 and NLR of 0.67. The sensitivity of the TB LAM Ag test was higher in HIV-positive participants: 57.1% compared to HIV-negatives (0.0%). The sensitivity of the TB LAM Ag test in participants with a CD4 cell count of 500 cells/mm^3^ and less was the same as with HIV-positive participants. The sensitivity of the TB LAM Ag test was 33.3% in both participants older than 45 years and those 45 years and younger. 

##### Combined Results from TB LAM Ag and Xpert MTB/RIF Ultra Assays

The diagnostic sensitivity, specificity, PPV, NPV, PLR, and NLR for the combined use of the TB LAM Ag and Xpert MTB/RIF ultra tests were the same as those of the Xpert MTB/RIF ultra test used alone. Similar to the overall performance, the performance was also the same as for the Xpert MTB/RIF ultra alone when results were stratified by HIV status, CD4 cell count, and age group.

##### Comparison of Diagnostic Efficiency

The sensitivity and NPV values of the Xpert MTB/RIF ultra test were higher than those of the TB LAM Ag test. The specificity and PPV of the TB LAM Ag test were higher than those of the Xpert MTB/RIF ultra test. The PLR of the Xpert MTB/RIF ultra test was higher than that of the TB LAM Ag test. The TB LAM Ag test had a higher NLR than the Xpert MTB/RIF ultra test. There was no improvement in diagnostic performance when the results of the TB LAM Ag test were combined with those of the Xpert MTB/RIF ultra test.

## 4. Discussion

The present study evaluated the diagnostic efficiency of TB LAM Ag and Xpert MTB/RIF Ultra while testing PCF samples from 44 patients with suspected TB pericarditis, 34.1% of whom had TBP confirmed by microbiological culture. The majority of participants with confirmed TBP rates were elderly, HIV-positive, and had CD4 cell counts of less than 200 cells/cm^3^.

The Xpert MTB/RIF Ultra test detected Mtb in 31.8% of PCF samples, which is comparable to rates from culture and higher than rates detected via PCF TB LAM Ag. The detection rate with Xpert MTB/RIF Ultra is greater than in Belgium (14.1%) [17], but lower than in China (83.7%) [18]. Though the observed rate is impressive given the short turnover for the results, it included two false positives, lowering the PPV to 85.7%. 

The reported PPV is lower than the PPV of the PCF TB LAM Ag test (100%). The value of PCF Xpert MTB/RIF Ultra is also lower than the PPV of 90% [17] and 98.1% [18] reported in previous studies. In addition, one of the studies reported 73 false positive results from 79 negative cultures [17] and the other 69 positive cases among 157 culture-negative cases [18]. According to Mekkaoui and colleagues, the majority of false positive cases were children and HIV-positive persons [17]. 

The two participants in the present study were old (average age = 56.5 years), one was HIV-positive with a low CD4 count (218 cells/cm^3^) but low viral load (61 copies/mL).

The false positives might be due to unresolved TB pericarditis, and because Xpert MTB/RIF Ultra identifies DNA of mycobacterium tubercle, it cannot distinguish between living and dead bacteria; both live and dead bacteria will result in a positive test with Xpert MTB/RIF Ultra. 

Other studies have found a few false positive Xpert MTB/RIF Ultra specificity in patients who had already received TB therapy [16,25,26]. The false positive outcomes were attributed to the Xpert MTB/RIF Ultra system’s enhanced technology [18]. The approach is intended to detect IS1081 and IS6110 targets as well as TB-specific areas in the rpoB gene [12]. The Xpert MTB/RIF Ultra, according to Mekkaoui and colleagues [17], might be effective when culture tests are positive but insufficient to eliminate TB with negative culture results.

Although the PCF Xpert MTB/RIF Ultra has a high specificity, it has a low sensitivity. Previous research found test sensitivities of 52.5% and 90%, respectively, and test specificities of 91.9% and 92%, respectively [17,18]. Just as with these previous studies, the sensitivity and specificity of PCF Xpert MTB/RIF Ultra obtained in the present study were 80% and 93.1%, respectively. The NPV value for PCF Xpert MTB/RIF Ultra was high (90%), validating the test’s ability to rule out TBP. Indeed, Xpert Ultra missed just three negative cases. Ageing, female gender, and immunosuppression were the traits of these patients with false negative results. The high NPV conforms to figures from prior investigations [17] which found an NPV of 99%, but it differs from the low NPV (19.5%) in another publication [18]. 

The accuracy of PCF Xpert MTB/RIF in defined subgroups showed an increased sensitivity in HIV-negative (100%) and elderly (83.3%) participants and a reduced specificity in participants with low CD4 cell count (80%).

The TB LAM Ag assay detected Mtb in 11.4% of cases, which is lower than the rates detected from culture and the Xpert Ultra test. This low percentage is attributable to the test’s failure to confirm 10 culture-positive cases. Although it properly identifies all true negatives, it fails to avoid false negatives, yielding an NPV of 74.4%, which is lower compared to the Xpert Ultra test. A far lower NPV (26.6%) was previously reported [23]. The NPV of 74.4% indicates that a TBP diagnosis should not be ruled out despite a negative TB LAM Ag test result.

The PCF TB LAM Ag test exhibited high specificity but low sensitivity, similar to the Xpert Ultra assay. Although the sensitivity (33.3%) was lower than that of Xpert Ultra, the specificity (100%) was higher. This is a recurring finding, as previous research found a similarly low sensitivity (11.6%) and high specificity (88%) for the PCF TB LAM Ag test in diagnosing TBP [23]. The test’s high specificity shows its ability to produce a true negative test result. Correctly so, there were no false positives, and the PPV for PCF TB LAM Ag was very high (100%), significantly higher than the PPV for Xpert Ultra results in the present study and the 72.7% from another South African population [23]. 

However, the TB LAM Ag test’s ability to diagnose TBP was influenced by the immunological state. Only HIV-positive patients tested positive for TB LAM Ag in this investigation. The sensitivity of the test was increased to 57.1% in HIV-positive participants as well as those with low CD4 cell counts. An association between the TB LAM Ag test and HIV was found in a previous investigation [23], where the assay was more sensitive to HIV-positive patients. Based on the findings from the present study, it is obvious that the TB LAM Ag test is superior in detecting TBP in HIV-positive individuals. Such findings are explained by the fact that the TB LAM Ag test detects *Mycobacterium tuberculosis* better in HIV-positive individuals, particularly the severely immune-compromised ones. The underlying mechanism via which the TB LAM can produce such positive results in HIV-infected populations has not been documented. This should be an aspect to consider for future investigation. The observed accuracy of TB-LAM in confirming TB in people living with HIV was produced from 16 studies which were reviewed by the World Health Organization [19]. The published policy guidance document supports the use of TB-LAM Ag assay with urine samples in this group of persons for early diagnosis of TB. In practice, TB LAM testing is now exclusively recommended to assist TB diagnosis in HIV-seropositive adults [27] in health systems around the world, including the South African health system [28]. However, despite the significant link observed from the findings of the present study, three of the ten false negatives were HIV-positive, with an average CD4 count of 277 cells/mm^3^. 

When the results of both tests were combined for sensitivity, specificity, PPV, NPV, PLR, and NLR, no change from the performance of Xpert MTB/RIF Ultra alone was observed. Thus, the TB LAM Ag test has no additive value when performing the Xpert MTB/RIF Ultra test in TBP diagnosis. Previous studies using PCF samples did not evaluate the efficiency of the combined test [17,18,23]. However, studies using urine samples showed improved efficiency for the combination with a marked increase in HIV-positive participants [29]. 

Looking at both findings, the detection rates for PCF Xpert MTB/RIF Ultra were greater than for the PCF TB LAM Ag test. The TB LAM Ag test’s sensitivity was 33.3%, compared to 80% for the Xpert MTB/RIF Ultra test. 

Our hypotheses regarding the difference in sensitivities between TB LAM Ag and Xpert MTB/RIF Ultra are multifaceted, and some may be because TBP can occur even when mycobacterium bacilli levels are low. TB LAM Ag may have failed to identify the lipoarabinomannan cell wall as readily as Xpert MTB/RIF Ultra, a PCR amplification test, and so the nucleic acid amplification would have made it simpler for Xpert MTB/RIF Ultra to detect TB even when the bacterial load is low.

The PLR of 0 for LAM indicates that it cannot be depended on to identify TBP, and the NLR of 0.67 for LAM indicates that it should rarely be used to rule out TBP. Comparable to these ratios, Pandie and colleagues found a PLR of 0.9 and NLR of 1.0 [23], showing that this test cannot be relied on often in diagnosing TBP. The PLR of 11.6 for PCF Xpert MTB/RIF Ultra indicates that it can be highly beneficial in detecting TBP, while its NLR of 0.21 indicates that it can occasionally be useful in ruling out a TBP diagnosis. Given these findings, the PCF Xpert MTB/RIF Ultra test outperforms the PCF TB LAM Ag test in identifying TBP. Nonetheless, in HIV-positive co-infected TBP patients, positive PCF TB LAM Ag findings should lead to treatment commencement, whereas negative results necessitate additional investigation. In the same light, a negative PCF Xpert MTB/RIF Ultra test does not rule out TBP. Further tests and therapies should be guided by the patient’s characteristics and presenting symptoms. 

The findings of this study are limited because the sample size was small; hence, some results may have occurred by chance. The small number of participants enrolled over 12 months confirms the rarity of this type of TB, which could be supported by the lack of recent statistics on TBP prevalence in South Africa and its provinces. Additional limiting aspects are that some of the subjects in the study were not microbiologically confirmed to be TB-positive; hence, the sensitivity and specificity of both the TB LAM Ag and the Xpert MTB/RIF Ultra results may have been underestimated or overestimated. 

## 5. Conclusions

PCF TB LAM Ag has better specificity, PPV, and NLR but lower sensitivity, NPV, and PLR compared to Xpert/MTB/RIF Ultra for the diagnosis of microbiologically confirmed TBP. PCF TB LAM Ag showed increased sensitivity in positive HIV status and low CD4 cell counts, but its sensitivity is independent of participants’ age. Xpert/MTB/RIF Ultra showed increased sensitivity in negative HIV status and older age but reduced specificity in low CD4 cell counts. PCF TB LAM Ag showed no additive value when performing Xpert/MTB/RIF Ultra for the diagnosis of TBP. A positive TB LAM Ag test in PCF samples necessitates immediate TBP treatment. If the TB LAM Ag test is negative, it should never be used to rule out TBP; nevertheless, it should be used in conjunction with the Xpert MTB/RIF Ultra and other PCF tests such as the ADA or interferon-gamma assay test to establish the absence of TBP. However, for the diagnosis of TBP, a TB LAM Ag test using PCF is recommended. 

## Figures and Tables

**Table 1:** Demographic and clinical features of patients suspected of tuberculous pericarditis.

Characteristics	n = 46
Age year; median (interquartile range)	48 (33.75–66.00)
Age year (min–max)	14–80
Male (n, %)	24 (52)
Female (n, %)	22 (48)
HIV-positive (n, %) N = 38	20 (52.6)
HIV-negative (n, %) N = 38	18 (47.4)
CD4 cell count; median (interquartile range)	253 (142.25–463.25)
Viral load; median (interquartile range)	2960 (922–121,350)

**Table 2:** TBP detection rates using culture results (n = 44).

Study Population	TBP, n (%)	Non-TBP, n (%)	*X*^2^ *p*-Value
All, n = 44	15 (34.1)	29 (65.9)	
Age group (years)			
≤45, n = 18	3 (16.7)	15 (83.3)	0.88
>45, n = 26	12 (46.2)	14 (53.8)	
HIV status			
Positive, n = 20	7 (35)	13 (65)	0.99
Negative, n = 18	6 (33.3)	12 (66.7)	
Not done, n = 6	2 (33.3)	4 (66.7)	
CD4 cell count categories (cells/mm^3^)			
<200, n = 4	4 (100)	0 (0.0)	0.008
200–500, n = 8	3 (37.5)	5 (62.5)	
>500, n = 2	0 (0.0)	2 (100)	
No data, n = 6	0 (0.0)	6 (100)	
Viral load categories (copies/mL)			
Below detectable limit, n = 4	3 (75)	1 (25)	0.28
Low, n = 1	0 (0.0)	1 (100)	
High, n = 8	2 (25)	6 (75)	
No data, n = 7	2 (28.6)	5 (71.4)	

**Table 3:** PCF Culture versus PCF Xpert MTB/RIF Ultra and PCF TB LAM Ag test results (n = 44).

Culture Results	Test Results	PCF Xpert MTB/RIF Ultra Results	PCF TB LAM Ag Results
Positive, n = 15	True positives	12	5
	False negative	3	10
Negative, n = 29	True negatives	27	29
	False positives	2	0

**Table 4:** Diagnostic performance of PCF TB LAM Ag and PCF Xpert MTB/RIF Ultra.

Performance Measurements	PCF TB LAM Ag	PCF Xpert MTB/RIF Ultra	Combined TB-LAM and Xpert Ultra
Sensitivity	33.3%	80%	80.0%
Specificity	100%	93.1%	93.1%
PPV	100%	85.7%	85.7%
NPV	74.4%	90.0%	90.0%
PLR	0.0	11.6	11.6
NLR	0.67	0.21	0.21

## Data Availability

The datasets generated and analyzed for this study are not publicly available due to participants’ privacy, but are available from the corresponding author upon reasonable request.
